# Assessment of Antipsychotic Medications on Social Media: Machine Learning Study

**DOI:** 10.3389/fpsyt.2021.737684

**Published:** 2021-11-18

**Authors:** Miguel A. Alvarez-Mon, Carolina Donat-Vargas, Javier Santoma-Vilaclara, Laura de Anta, Javier Goena, Rodrigo Sanchez-Bayona, Fernando Mora, Miguel A. Ortega, Guillermo Lahera, Roberto Rodriguez-Jimenez, Javier Quintero, Melchor Álvarez-Mon

**Affiliations:** ^1^Department of Medicine and Medical Specialities, University of Alcala, Alcala de Henares, Spain; ^2^Ramón y Cajal Institute of Sanitary Research (IRYCIS), Madrid, Spain; ^3^Department of Psychiatry and Mental Health, Hospital Universitario Infanta Leonor, Madrid, Spain; ^4^Cardiovascular and Nutritional Epidemiology, Institute of Environmental Medicine, Karolinska Institute, Stockholm, Sweden; ^5^IMDEA-Food Institute, CEI UAM+CSIC, Madrid, Spain; ^6^IBM Data and AI Expert Labs and Learning, London, United Kingdom; ^7^Department of Psychiatry and Clinical Psychology, University of Navarra Clinic, Pamplona, Spain; ^8^Navarra Institute for Health Research (IdiSNA), Pamplona, Spain; ^9^Hospital Universitario 12 de Octubre, Unidad de Cáncer de Mama y Ginecológico, Madrid, Spain; ^10^Department of Legal and Psychiatry, Complutense University, Madrid, Spain; ^11^Department of Psychiatry, University Hospital Principe de Asturias, Alcalá de Henares, Spain; ^12^CIBERSAM (Biomedical Research Networking Centre in Mental Health), Madrid, Spain; ^13^Instituto de Investigación Sanitaria Hospital 12 de Octubre (imas 12), Madrid, Spain; ^14^Universidad Complutense de Madrid (UCM), Madrid, Spain; ^15^Service of Internal Medicine and Immune System Diseases-Rheumatology, University Hospital Príncipe de Asturias (CIBEREHD), Alcalá de Henares, Spain

**Keywords:** psychosis, psychiatry, neuropsychopharmacology, antipsychotics, machine learning, artificial intelligence, pharmacoepidemiology

## Abstract

**Background:** Antipsychotic medications are the first-line treatment for schizophrenia. However, non-adherence is frequent despite its negative impact on the course of the illness. In response, we aimed to investigate social media posts about antipsychotics to better understand the online environment in this regard.

**Methods:** We collected tweets containing mentions of antipsychotic medications posted between January 1st 2019 and October 31st 2020. The content of each tweet and the characteristics of the users were analyzed as well as the number of retweets and likes generated.

**Results:** Twitter users, especially those identified as patients, showed an interest in antipsychotic medications, mainly focusing on the topics of sexual dysfunction and sedation. Interestingly, paliperidone, despite being among one of the newest antipsychotics, accounted for a low number of tweets and did not generate much interest. Conversely, retweet and like ratios were higher in those tweets asking for or offering help, in those posted by institutions and in those mentioning cognitive complaints. Moreover, health professionals did not have a strong presence in tweet postings, nor did medical institutions. Finally, trivialization was frequently observed.

**Conclusion:** This analysis of tweets about antipsychotic medications provides insights into experiences and opinions related to this treatment. Twitter user perspectives therefore constitute a valuable input that may help to improve clinicians' knowledge of antipsychotic medications and their communication with patients regarding this treatment.

## Introduction

Schizophrenia is a psychiatric disorder characterized by a multidimensional psychopathology that includes positive, negative and mood symptoms, as well as cognitive impairment. Additionally, it is commonly associated with impairments in social and occupational functioning (1). Schizophrenia and psychotic disorders are among the world's leading causes of disability (2). However, evidence-based psychosocial interventions, in conjunction with pharmacotherapy, can help patients achieve recovery (3, 4).

Antipsychotic medications are the first-line treatment for psychosis-related disorders and have been shown to be effective in treating associated symptoms and behaviors (5). First- and second-generation antipsychotic medications are comparable in clinical efficacy, with the exception of clozapine, which demonstrates the best results toward treatment-resistant schizophrenia (6, 7). However, they differ from one another in other characteristics such as dosing, the route of administration, the side effects and the cost (8). All these factors influence the selection of the antipsychotic drug to be used, as well as adherence to a medication regimen, ultimately affecting a medication's effectiveness. For example, weight gain, diabetes, and dyslipidemia are more often associated with second-generation antipsychotics (SGAs), while first-generation antipsychotics (FGAs) generally have a greater risk of extrapyramidal side effects (8, 9).

In patients with a poor response to medication or repeated relapses, non-adherence should be considered (10, 11). Side effects, along with a limited awareness of one's schizophrenia and the need for treatment, are frequently the primary cause of non-adherence, and thus need to be addressed (3). However, many patients hide these issues from their doctor, with the reasons for doing so poorly understood yet nevertheless significant, such as the feeling of shame in disclosing these aspects or the fear of being judged (11).

Traditional research on patients' experiences to treatment has principally relied on surveys or interviews (12–14). However, the analysis of social media posts has emerged as an important tool capable of gathering a more comprehensive understanding of those factors involved in the therapeutic process and in the experience of the disease (15, 16). It also allows for greater insights into the opinions of others apart from just the patients themselves, thereby providing additional data from a wider range of perspectives, including those patients that are reluctant to visit a medical professional (15–17). In addition, social media conversations are generated within a more casual and spontaneous environment than those that take place during a medical appointment; thus, they may be more likely to reflect true beliefs (18–20).

Furthermore, numerous studies have also demonstrated that individuals living with schizophrenia use popular social media platforms at comparable rates to the general population (21). In addition, individuals with mental illness appear to use social media to share their illness experiences or seek advice from others with similar conditions (21). However, less is known about whether people with psychosis talk about antipsychotic medication experiences over social media. Thus, our aims were to (1) investigate the frequency of online communications about antipsychotic medications among Twitter users; (2) characterize the type of users participating in these conversations; (3) determine the main thematic content of Twitter posts and the interest they generated; and (4) analyze references to specific antipsychotic medications.

## Methods

### Search Strategy and Collection of Twitter Data

In this observational quantitative and qualitative study, we focused on searching for tweets that referred to antipsychotics. We collected all posted tweets using the following list of keywords: amisulpirida, solian, haloperidol, haldol, pimozida, orap, sulpirida, dolmatil, flufenacina, modecate, sulpirirda, dogmatil, clorpromacian, largactil, levomepromazine, sinogan, aripiprazol, abilify, clozapine, leponex, nemea, clozaril, olanzapina, zyprexa, paliperidone, invega, quetipiana, seroquel, risperidone, risperdal, brexpiprazole, rexulti, loxapina, adasuve, lurasidone, latuda, ziprasidone, geodon, and zeldox. We selected keywords according to the most frequently prescribed antipsychotics in Europe and the United States. We have included both generic names and brand names in the selection. We grouped the keywords into 13 categories, with each category corresponding to a different antipsychotic medication, thus allowing for greater ease when comparing one medication with another. Antipsychotic medications were referred to by either their generic name or by their brand name.

The inclusion criteria for tweets were: (1) Being public; (2) Containing any of the previously mentioned keywords; (3) Being posted between January 1st 2019 and October 31st 2020 and; (4) Containing text in English or Spanish. A 22 month period was chosen to avoid any potential bias in the content of the tweets. For instance, we wanted to be sure to prevent content from being affected by the season of the year, a particular event, or a special circumstance (publication of any relevant scientific article related to any of the antipsychotic medications being analyzed, the mention of or a relationship to any medical, psychiatric or pharmacological conferences, etc.). In addition, we obtained the number of retweets and likes each tweet generated as an indicator of user interest on a given topic, the date and time of each tweet, a permanent link to the tweet and each user's profile description. Tweet Binder, the search engine we employed, allows access to 100% of all public tweets that match certain criteria.

### Content Analysis Process

All 30,603 retrieved tweets were included in the dataset. First, we randomly selected 1,500 of the tweets to be considered for content analysis. Secondly, we created a codebook based on our research questions, our previous experience in analyzing tweets, and what we determined to be the most common tweet themes (22–25). Third, JG and LA analyzed 300 tweets separately to test the suitability of the codebook. Discrepancies were discussed between the raters and with another two authors (MAAM and MAM), and after revising the codebook the raters then proceeded to manually code the remaining 1,200 tweets.

Tweets were categorized as classifiable or unclassifiable. We considered a tweet as non-classifiable when its content did not provide enough information or if it was written in a language other than English or Spanish. Each of the tweets considered classifiable was rated according to the area of clinical interest mentioned or discussed in the text of the tweet, such as quality of life, mood and anxiety, sedation, metabolic disturbances and extrapyramidal symptoms, sexual dysfunction, and cognitive complaints. In addition, tweets with non-medical content were rated as commercial, economic, ask/offer help, trivialization, or non-specific. We classified as trivialization those tweets that included mockery or joking, associated treatment with undesirable attributes or associated treatment with grossly inaccurate stereotypes. Moreover, the following characteristics were also analyzed: if the tweet included a link to a health care provider (either a hospital, health institution, university, or pharmaceutical company), if it mentioned a scientific article or specific aspects of posology, or if it expressed a personal opinion, mentioned a famous person or stated the use of an antipsychotic to treat a particular disorder. Finally, users were classified into 5 categories: patient, relative or friend of a patient, health professional, health institution, and Twitter-user interaction (when at least two different users participated in a conversation but were classified into different categories). We determined the nature of the users that posted tweets according to the information available: content of the tweet (use of pronouns, disclosure of personal or family experiences, professional information provided, etc.), user profile description or Twitter handle (this section was especially informative in the case of health institutions since the majority refer to their social media accounts in their profile description or their Twitter handle). In those cases in which the nature of the user was not possible to know, they were considered as indeterminate.

The coding categories used were not mutually exclusive. In the case of finding content that was repeated exactly or posted almost identically in different tweets, those postings were classified in the same way as the first tweet encountered.

### Multilingual Machine Learning Classifier

The goal of the initial tagging of 1,500 tweets was to provide data to train, test, and validate Machine Learning classifiers so that any extracted tweet classifications could be inferred. To train the classifiers, the transformer multilingual model xlm-roberta neural net was used in conjunction with a “k-train” library to deploy it (26, 27). The following additional features were generated to improve understanding of the selected set: the number of tokens that the sentence contained, the total length of the tweet in terms of characters, the language of the tweet and the extracted hashtags from the tweet. Additionally, on order to improve the Machine Learning classifier performance, we generated a clean text that took any mentions (@) and hyperlinks out of the tweet so that it became more readable.

Out of the 1,500 manually labeled tweets, we reserved 10% to use as a blind set for model validation so that the setup we employed for training the classifier constituted 80% training and 20% validation. Initially, training was done on the classifiable feature and then out of those tweets that were labeled classifiable, the remaining classifications were trained. The weighted average F1 score of the training validation against the blind dataset was above 0.80 in all cases except for the User and Interest categorization, which was slightly lower.

These analyses were performed using Python 3.7 and the libraries “pandas,” “numpy,” “json,” and “k-train.”

### Ethical Considerations

This study received the approval of the University of Alcala Research Ethics Committee and was compliant with the research ethics principles of the Declaration of Helsinki (7th revision, 2013). However, this study did not directly involve human subjects nor include any intervention but instead used only publicly available tweets. Nevertheless, we have taken care not to reveal any usernames and to avoid citing any tweets that could reveal them.

### Statistical Analysis

The frequency distribution (percentage) of tweets, retweets, and likes according to certain characteristics of each tweet, such as the content included or the antipsychotic drug mentioned, were displayed across several figures and tables. Because the sample of tweets corresponds to all tweets from the selected period, and are not just a representative sample of these tweets, the causal inference *p*-values do not apply. The accuracy of the different tweet distributions obtained from the Multilingual Machine Learning models is reflected by the weighted average F1 score (a combination of precision and recall; the closer the score is to 1, the less possibility of classification error). As well, retweet-to-tweet and like-to-tweets ratios according to user type, the area of clinical interest and non-medical aspects were calculated.

According to the content of the tweet, we used simple logistic regressions to calculate the probability of retweeting or liking a tweet. These results were presented as an odds ratio (OR) with 95% Confidence Intervals (CI). These analyses were conducted with the software packages STATA v16 (StataCorp) and MS Excel.

## Results

### Sedation and Sexual Dysfunction Are the Most Common Areas of Clinical Interest in Antipsychotic Related Tweets

For this work, we collected 30,603 tweets including the keywords antipsychotic medications from January 1st 2019 to October 31st 2020. According to the inclusion criteria of the codebook, a total of 22,092 tweets were considered classifiable.

**Figure 1:**
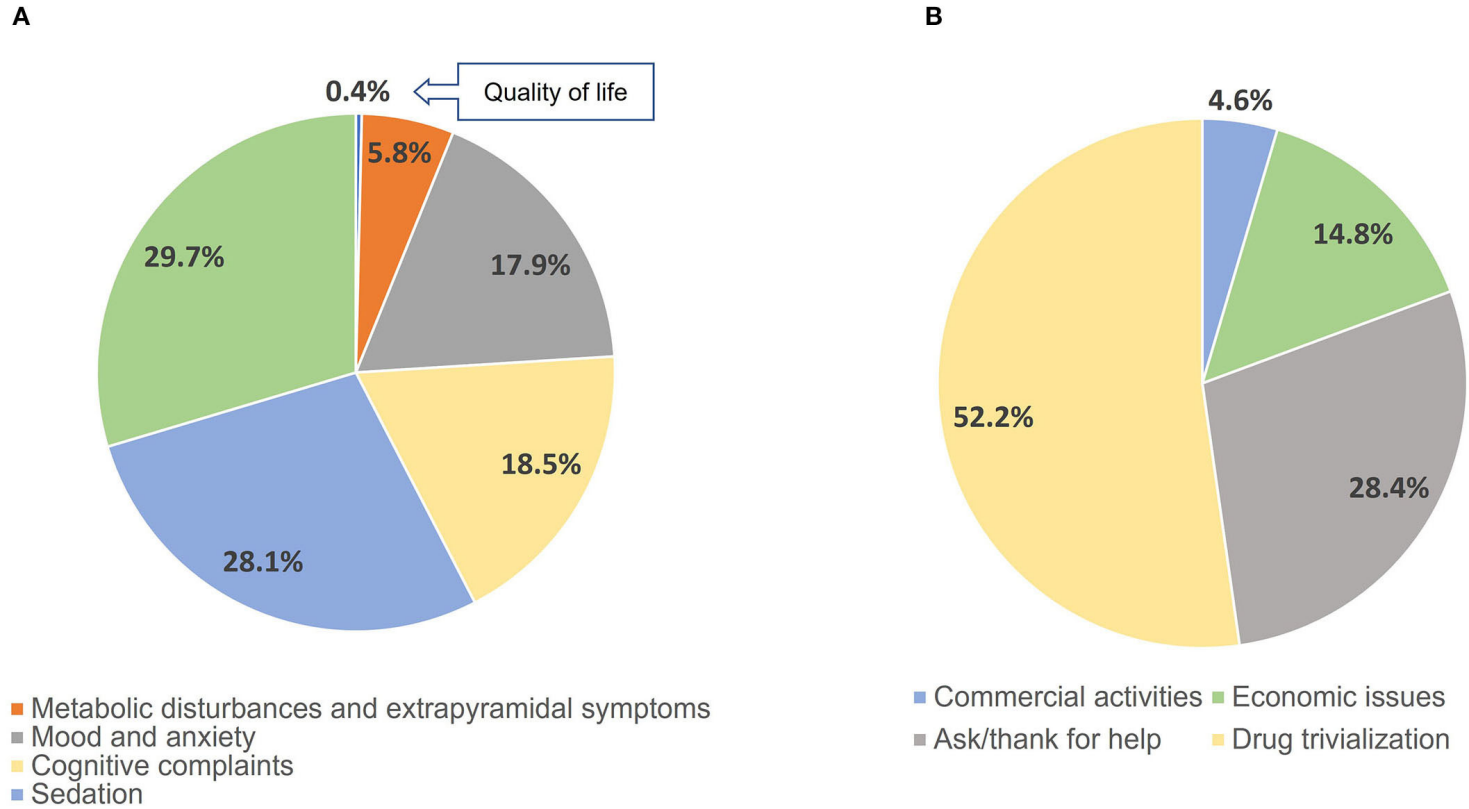
Different percentages (%) of tweets according to areas of clinical interest **(A)** and non-medical aspects **(B)**.

First, we analyzed the content of those tweets referencing the specific areas of clinical interest studied (Figure 1A), with 5,415 out of the 22,092 tweets analyzed featuring related content. There was a significant difference in distribution (*P* < 0.05) of the number of tweets between the different categories studied, the predominant majority being related to sexual dysfunction and sedation. Additionally, there was a lower frequency of tweets related to cognitive complaints and mood or anxiety. Those related to metabolic disturbances or extrapyramidal symptoms represented the minority of tweets.

We also analyzed the content of those tweets including no medical aspects of antipsychotic medications (8,517 tweets) and we classified them according to four categories: commercial activities, economic issues, ask for or offer help and trivialization) (Figure 1B). We found a significant distinction in the number of tweets between these four categories with the highest frequency of postings related to drug trivialization followed by those asking for or offering help (*P* < 0.05). Those related to commercial activities and economic issues represented the minority of tweets.

Further investigating certain tweet characteristics, we found that 18.87% of the 22,092 tweets studied included a link to a health care provider (either a hospital, health institution, university, or pharmaceutical company), 10.55% referenced a scientific article and 10.44% were related to specific aspects of posology. Moreover, 14.1% of the tweets included a personal opinion while 6.4% mentioned a famous person. Lastly, 17.7% mentioned the use of antipsychotics for treating a particular psychiatric disorder.

### Patients Are the Most Active Twitter Users With a Differential Pattern of Areas of Interest in Antipsychotic Related Conversations

From investigating the types of users that posted tweets, 7,188 tweets were posted by users identified as patients (72.1%), health institutions (17.2%), patients' friends or relatives (7.0%), and health care professionals (3.7%). Seven thousand six hundred and fifty-five tweets were interactions between two or more Twitter users. From the remaining 4,465 tweets, the users were considered unclassifiable.

**Figure 2:**
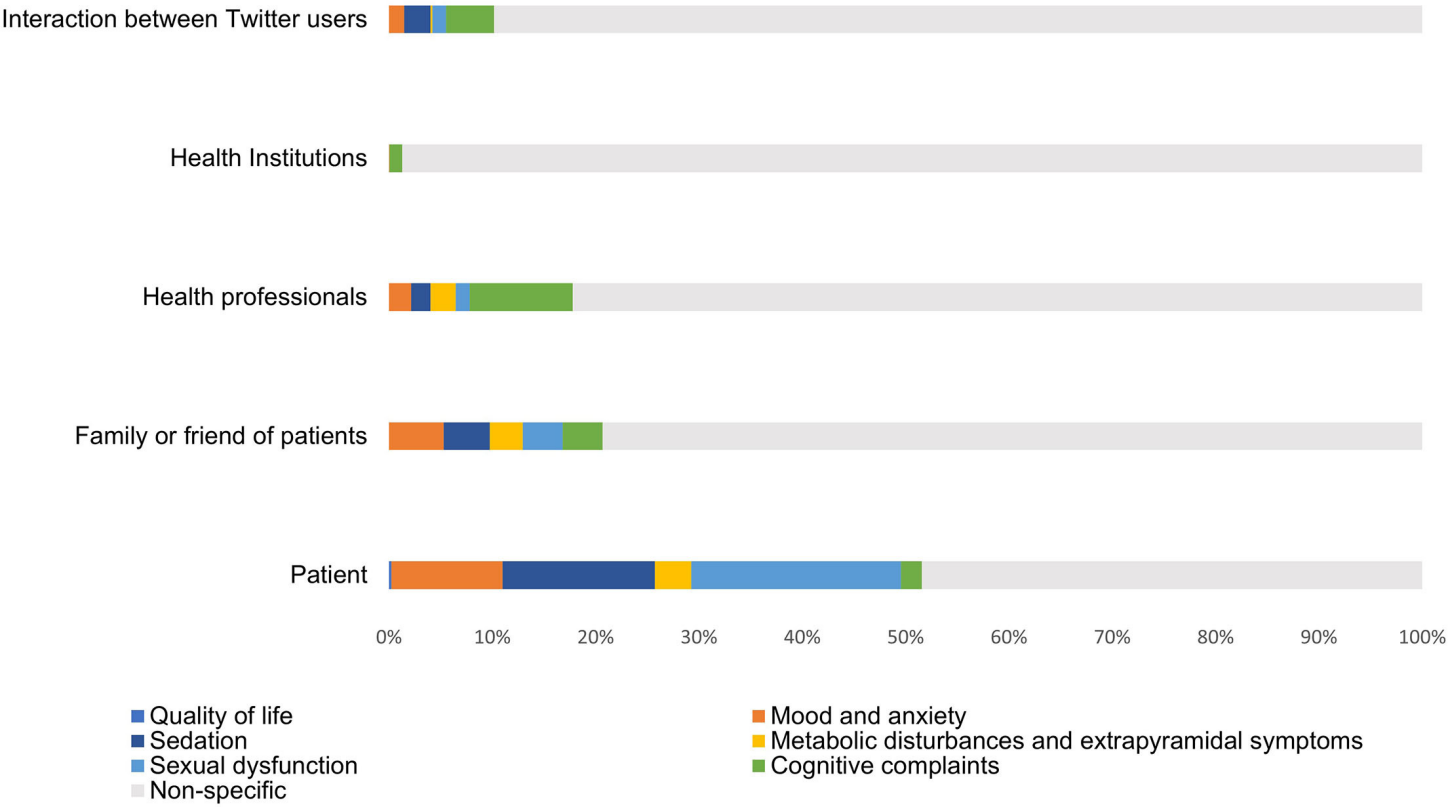
Areas of clinical interest related to antipsychotic medications by the type of user posting. Percentages (%) were calculated with respect to the total number of tweets posted by each type of user.

Subsequently, we investigated areas of clinical interest in those tweets posted by different types of users, finding significant differences between them (*P* < 0.05) (Figure 2). Patients, for example, focused the content of their tweets on the specific clinical aspects of antipsychotic medications, specifically sexual dysfunction and sedation. In contrast, almost all of the tweets posted by health institutions concerned non-specific issues. However, the distribution of postings related to areas of clinical interest stemming from patients' friends or relatives, health professionals and Twitter interactions was similar, with a predominance centered on cognitive complaints.

### Tweets Asking for or Offering Help, Referring to Cognitive Impairment or Being Posted by Health Institutions Generated the Greatest Interest

We investigated the interest generated by those tweets related to antipsychotic medications by quantifying the number of retweets and likes generated by each tweet. In doing so, we found that the probabilities of a tweet being retweeted or liked were distinct between the different categories of users. Tweets posted by health institutions accumulated the highest median of retweets per tweet, which was three times higher than that observed in tweets posted by health care professionals or patients' relatives and friends (Table 1). Not insignificantly, those tweets posted by patients obtained the lowest median of retweets per tweet. By contrast, when we analyzed the probability of tweets receiving likes, we found a noticeable similarity among the different types of users. Nevertheless, tweets posted by health professionals obtained twice as many likes as those posted by patients, health institutions and patients' relatives (Table 1).

**Table 1:** Retweet-to-tweet and like-to-tweet ratios per type of user.

**Type of user**	**Retweet/tweet ratio**	**Like/tweet ratio**
Patient	0.15	2.10
Family or friend of patients	5.57	2.41
Health professionals	5.37	5.41
Health institutions	17.60	2.53
Interaction between Twitter users	1.53	6.60
Non-specific	2.00	5.37

Furthermore, we investigated the number of likes and retweets generated by each area of clinical interest (Table 2). Tweets related to cognitive complaints were clearly those with the highest ratios of being retweeted and liked, followed by those referencing mood and anxiety and sedation. Finally, those tweets concerned with quality of life, metabolic disturbances and extrapyramidal symptoms, and sexual dysfunction had a similar average number of retweets and likes per tweet. Additionally, we investigated the probability of a tweet of being retweeted or liked depending on non-medical aspects. We found that tweets asking for or offering help were clearly retweeted more than those either referring to commercial or economic issues and trivialization toward a certain drug. That being said, tweets related to drug trivialization still received the most likes (Table 3).

**Table 2:** Retweet-to-tweet and like-to-tweet ratios per area of clinical interest.

**Area of clinical interest**	**Retweet/tweet ratio**	**Like/tweet ratio**
Quality of life	0.10	1.70
Mood and anxiety	1.02	6.62
Sedation	0.31	2.91
Metabolic disturbances and extrapyramidal symptoms	0.16	1.91
Sexual dysfunction	0.12	1.96
Cognitive complaints	6.24	18.84

**Table 3:** Retweet-to-tweet and like-to-tweet ratios per non-medical aspects.

**No medical content**	**Retweet/tweet ratio**	**Like/tweet ratio**
Commercial activities	0.77	4.72
Economic issues	1.14	3.10
Ask for or offer help	15.69	2.54
Trivialization	0.99	5.99

**Figure 3:**
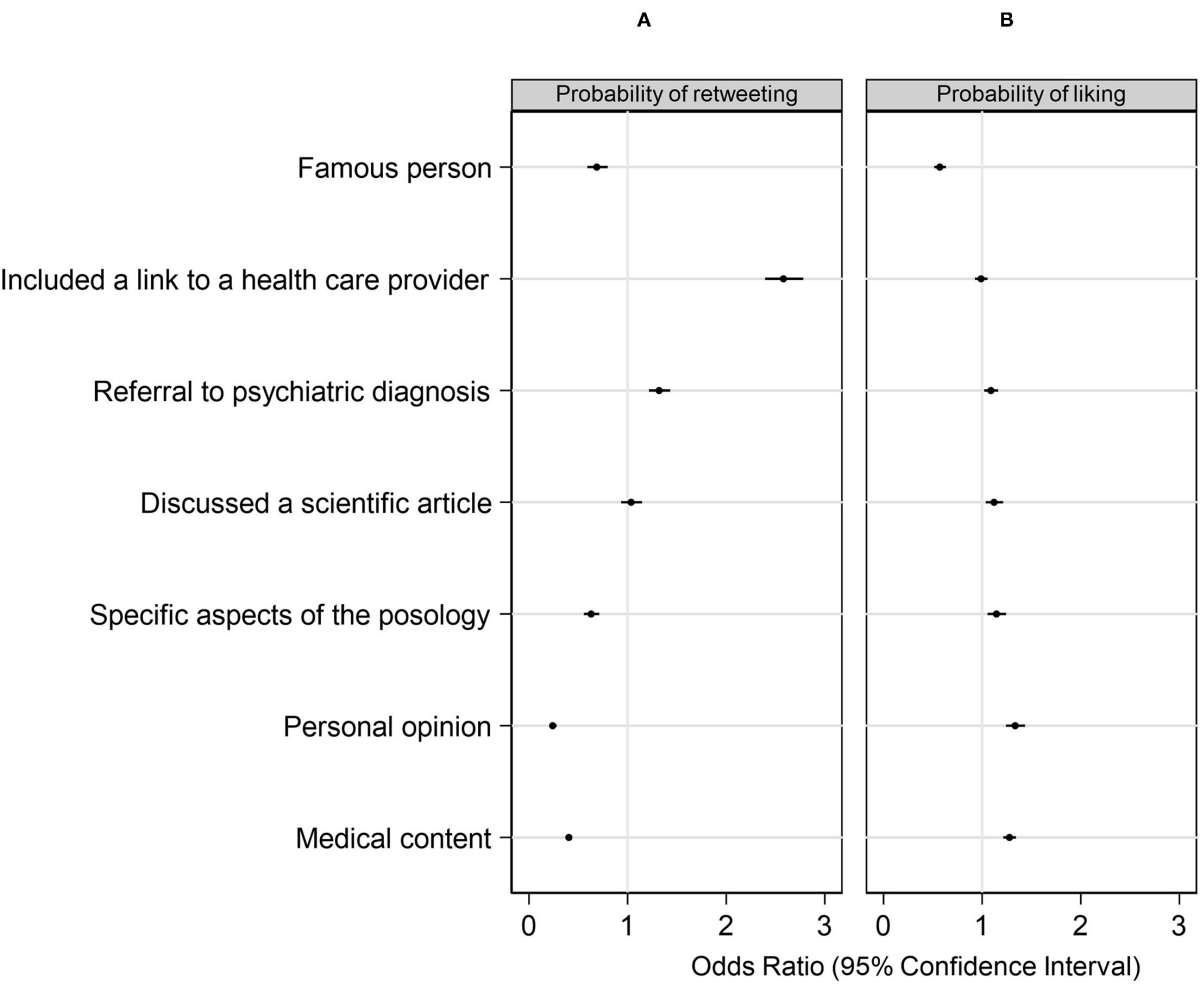
ORs and 95% CIs for retweeting **(A)** and liking **(B)** a tweet according to specific characteristics.

Finally, we assessed which characteristics were more associated with engagement (Figure 3). We found that those tweets including a link to a health care provider had almost three times greater odds of being retweeted than those tweets that did not include such a link. As well, tweets that referred to a psychiatric diagnosis had a greater probability of being retweeted than those tweets that did not. On the other hand, tweets that mentioned a famous person or specific aspects of posology, or that expressed a personal opinion, had lower probabilities of being retweeted. By contrast, the probabilities of a tweet being liked maintained a different pattern even though most of the associated characteristics being analyzed had a modest effect on the probability of a tweet being retweeted or liked.

### The Frequency of Tweets Related to Antipsychotic Medications Was Heterogeneous

**Figure 4:**
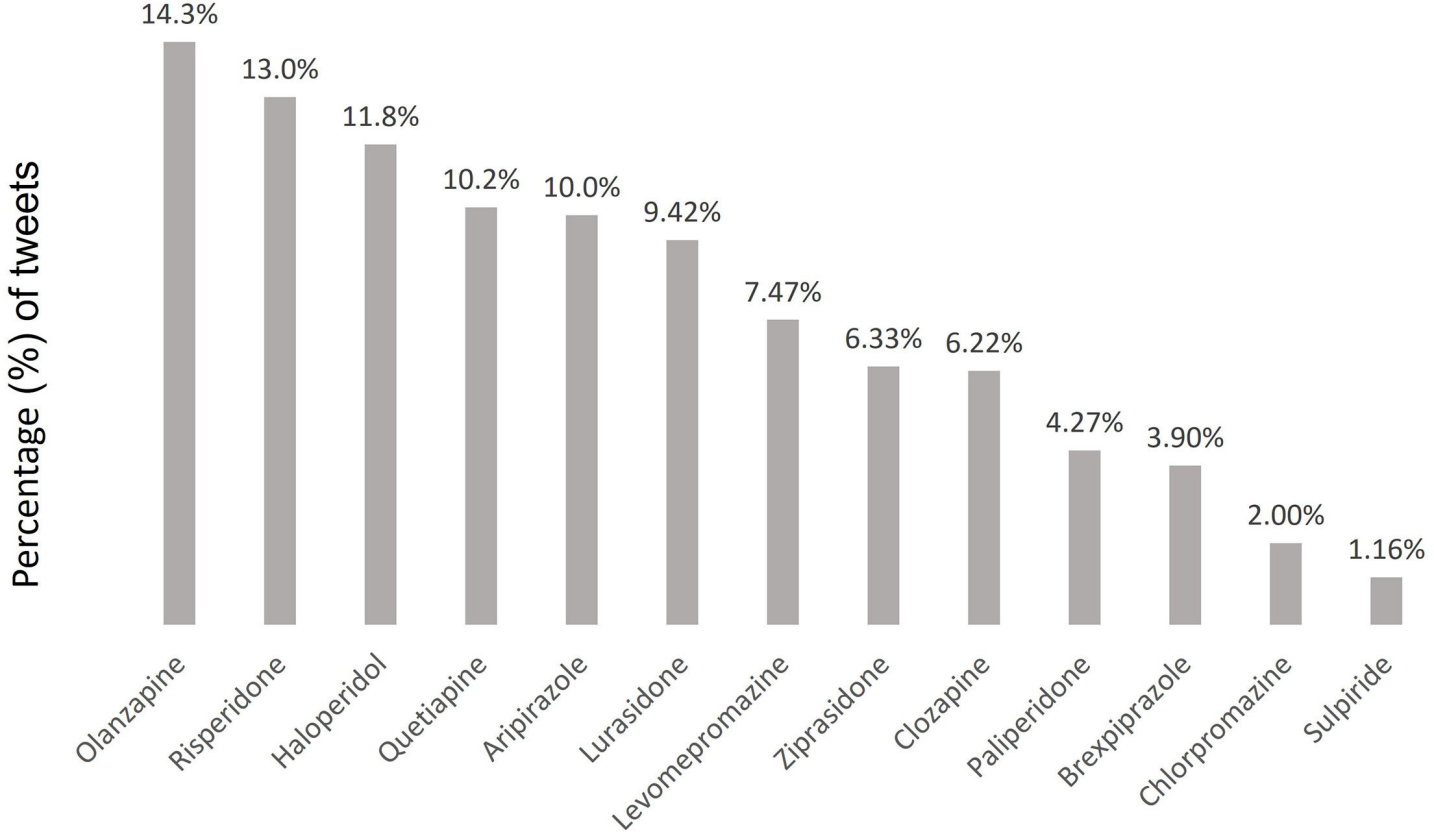
Distribution of tweets by antipsychotic medications.

Of the 22,092 tweets included in our research, we investigated those specifically related to each of the 13 antipsychotic medications studied. The number of mentions of each drug followed a heterogeneous pattern of distribution, ranging from 14.3% for olanzapine to 1.2% for sulpiride (Figure 4). Of note, aripiprazole and paliperidone, which are among the newest antipsychotic medications, only accounted for 15.0% of the total number of tweets.

**Figure 5:**
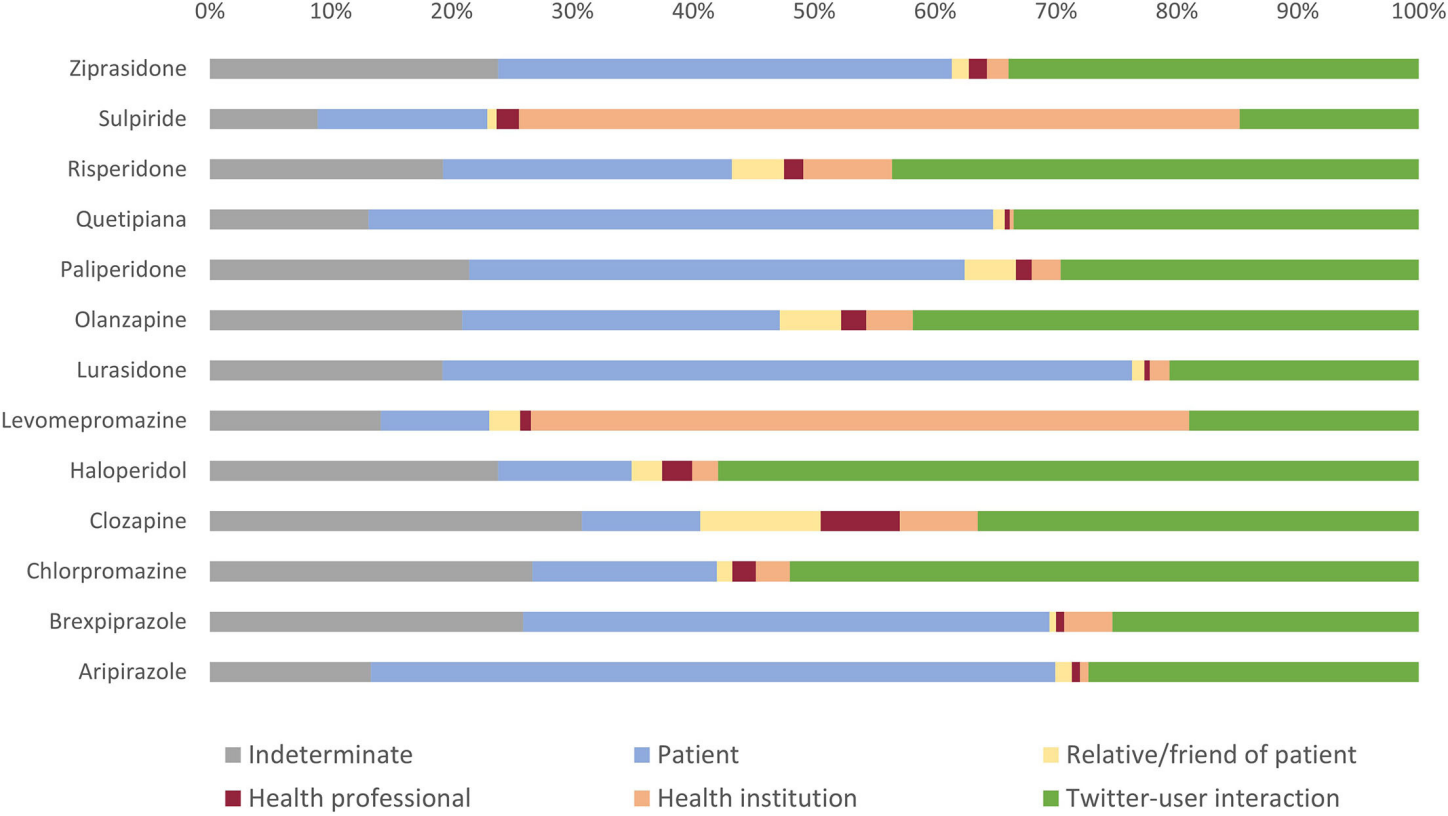
Tweets related to each antipsychotic medication by the type of user posting. Percentages (%) were calculated with respect to the total number of tweets generated on each antipsychotic medication.

We also analyzed the type of user who posted tweets related to antipsychotic medications. For example, the percentage of tweets posted by users identified as patients was greater in lurasidone (57.0%), aripiprazole (56.6%), quetiapine (51.7%), brexpiprazole (43.6%), and paliperidone (41.0%) (Figure 5). The percentage of tweets posted by users identified as friends and relatives of patients or by health professionals was low overall, with the exception of clozapine (10.0 and 6.6%, respectively). Tweets posted by health institutions held higher percentage totals when related to sulpiride (59.6%) and levomepromazine (54.5%) but constituted a total below 10% when referencing all other drugs. Lastly, Twitter user interaction represented the category with the highest percentage of tweets related to haloperidol (58.0%), chlorpromazine (52.2%), risperidone (43.6%), or olanzapine (41.8%).

### Most Recent Medications Did Not Attract Much Attention Among Twitter Users

**Figure 6:**
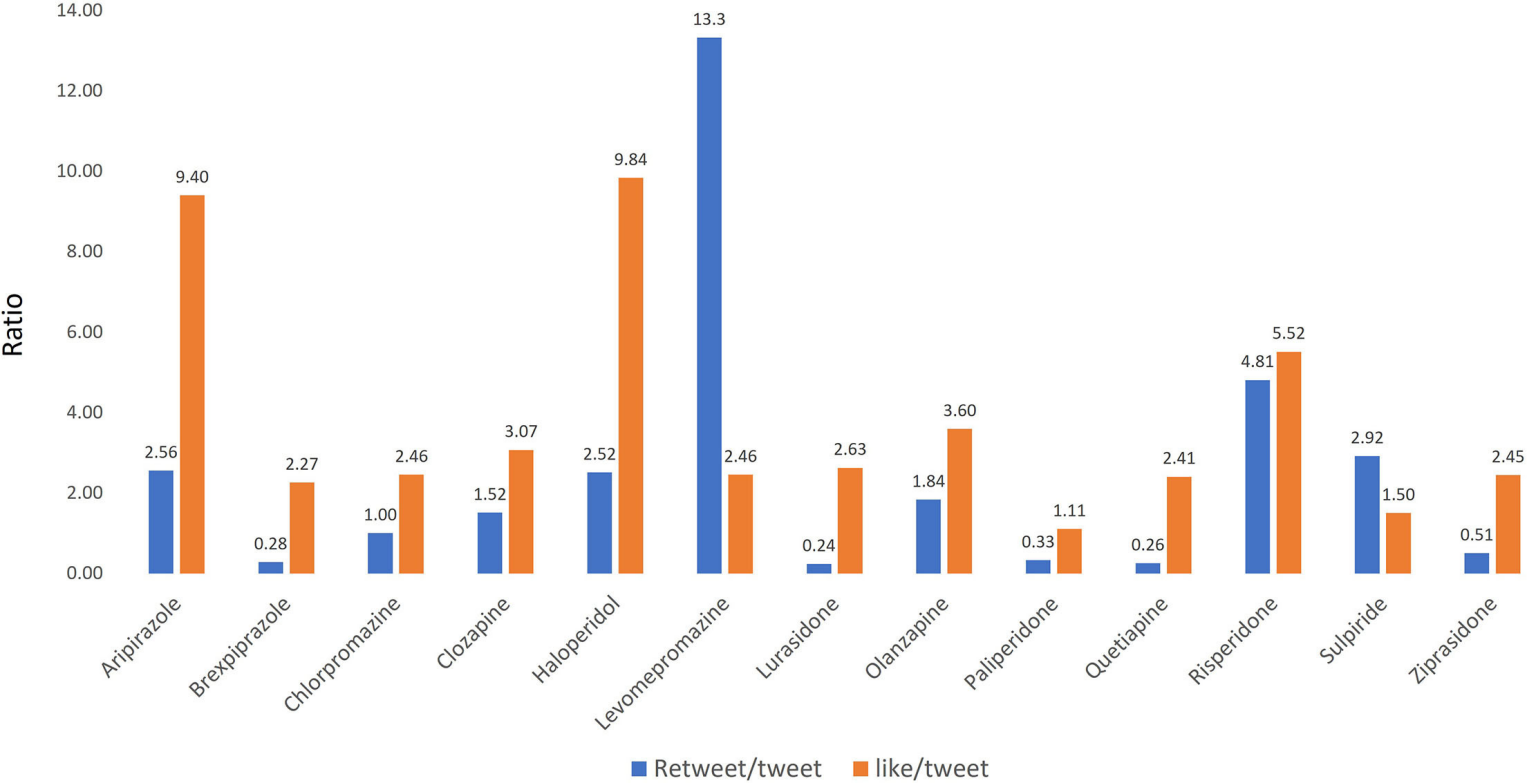
Retweet-to-tweet and like-to-tweets ratios per each antipsychotic medication.

We measured the number of retweets and likes generated by those tweets related to each of the antipsychotic medications studied. We found that the retweet-to-tweet and like-to-tweet ratios between the different drugs were distinct (Figure 6). For instance, levomepromazine was the drug with the highest retweet-to-tweet ratio (13.3), whereas haloperidol and aripiprazole were the drugs with the highest like-to-tweet ratios (9.8 and 9.4, respectively). Moreover, we found that brexpiprazole, lurasidone, paliperidone, quetiapine, and ziprasidone generated a retweet-to-tweet ratio below 1.

**Figure 7:**
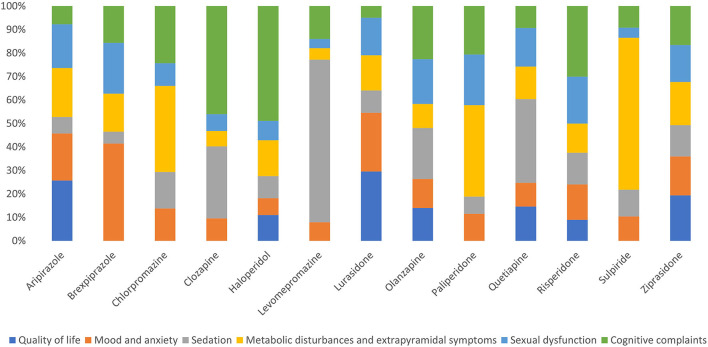
Tweets related to each antipsychotic medication by areas of clinical interest. Percentages (%) were calculated with respect to the total number of tweets generated on each antipsychotic medication.

We also analyzed the distribution of tweets referencing an antipsychotic medication according to areas of clinical interest (Figure 7). Almost 60% of the tweets referring to quality of life mentioned aripiprazole or lurasidone, whereas none of the tweets mentioning sulpiride, paliperidone, levomepromazine, clozapine, chlorpromazine, or brexpiprazole addressed issues related to quality of life. In regards to mood and anxiety, we found that this type of content was minimal in tweets mentioning chlorpromazine, clozapine, haloperidol, levomepromazine, paliperidone, or sulpiride. A similar pattern was observed in tweets discussing issues related to sedation. Interestingly, tweets mentioning metabolic disturbances and extrapyramidal symptoms had a similar pattern of distribution to those tweets discussing sexual dysfunction, with aripiprazole, lurasidone, and quetiapine accumulating most of the tweets. Finally, those tweets referencing issues related to cognitive impairment were also distributed heterogeneously among the different drugs; in this case, haloperidol, olanzapine, and risperidone received the majority of tweets.

## Discussion

In this study, we have found that Twitter users show a great interest in antipsychotic medications focused on sexual dysfunction and sedation, especially those users identifying themselves as patients. Tweets asking for or offering help and those posted by institutions obtained the highest retweet-to-tweet ratio, whereas tweets discussing cognitive complaints obtained the highest like-to-tweet ratio. Moreover, health professionals did not command a strong presence in driving Twitter conversations with regards to antipsychotics, nor did medical institutions. In addition, tweets referring to the trivialization of certain medications was frequently observed. Interestingly, paliperidone, despite being among one of the most recent drugs launched on the market, nevertheless has failed to generate much interest among Twitter users, accounting for only a small number of tweets.

Furthermore, the medical treatment of patients with chronic diseases has become an increasing challenge to society, with several factors involved in patient outcomes (28). These are the efficacy and side effects of medications, access to medical information and social considerations toward a particular disease and its treatment, all of which serve to influence patients' attitudes in regards to treatment (29, 30). Thus, identifying both patients' and the public's areas of concern about diseases and their treatment in general, along with mental health conditions in particular, is extremely relevant for improving clinical outcomes (31). Correspondingly, the analysis of social media platforms such as Twitter is a recognized tool to explore societal opinions of chronic health conditions (32). Unfortunately, schizophrenia and psychosis-related disorders are frequently mischaracterized pejoratively over Twitter (33–36). In fact, a previous study found that school shooting was among the hashtags most frequently associated with psychosis, which shows that there are still many people who mistakenly associate psychosis with aggressiveness or violence (31). This association of negative hashtags does not occur in other physical illnesses such as breast cancer or diabetes, nor in other mental illnesses such as Hikikomori (37). Furthermore, in this study, we have found that antipsychotic medications are often trivialized in tweets. Collectively, Twitter data shows that patients don't just suffer from psychosis as antipsychotic treatments are also the targets of negative feelings and judgments by Twitter users, further demonstrating the persistence of social stigma toward schizophrenia and its treatment (38–41). This experience of stigmatizing attitudes against people with schizophrenia may as a result be internalized, leading to “self-stigma” that contributes to poor patient adherence to treatment (42, 43).

A comparative analysis of the prevalence of psychosis and the number of tweets posted related to antipsychotic medications, contrasted with those referencing treatment received for other chronic diseases, supports the notion of considerable interest in antipsychotic medications among the Twitter community (44). Interestingly, our results show that patients with schizophrenia are the most common users posting content about antipsychotic medications. These results contrast with the findings reported in research describing other medical diseases mentioned on Twitter (45). Several reasons may explain this higher use of Twitter by people living with schizophrenia. First, the anonymity provided by this social media platform may favor use by persons suffering from stigmatization. Secondly, the difficulty in personal communication that these patients exhibit is mitigated through their online interactions. Third, easy accessibility to Twitter and facility in posting content may also be involved in the use of this platform by people living with schizophrenia who otherwise might not possess the willingness and confidence to engage with others in person.

In addition, social media appears to be especially valuable for monitoring the treatment experiences of individuals with psychotic related disorders who frequently use online platforms to engage with people facing similar struggles (46, 47). Our data shows that patients posted tweets related to specific areas of clinical interest stemming from antipsychotic treatment, namely sedation, sexual dysfunction, mood, and anxiety. In fact, sexual dysfunction is common with antipsychotic treatment although its frequency varies among studies. For example, a 2011 meta-analysis found that around half of those patients taking clozapine, olanzapine, and risperidone reported sexual side effects, while those using aripiprazole, quetiapine, and ziprasidone experienced lower rates (48). However, other clinical trials have reported even lower frequencies of sexual dysfunction. These differences may be due in part to patients' reluctance to report sexual side effects during in-person interviews (49). In contrast, from our Twitter data, sexual dysfunction was one of the most discussed topics, which reinforces the notion of the platform as a resource that, as a result of its particular characteristics, can better serve to facilitate patients' disclosure of sensitive, potentially embarrassing information. The meta-analysis indicated above also found that among patients who experienced sexual dysfunction secondary to an antipsychotic medication, switching to aripiprazole was found to be the most common solution (50). Interestingly, aripiprazole was the antipsychotic that accounted for the most tweets related to sexual dysfunction.

Patients with schizophrenia have higher rates of depressive and anxiety disorders than the general population (51). Noticeably, this issue has been discussed more frequently by the patients themselves than by relatives or friends, health professionals and health institutions. This is probably due to the fact that patients are the ones most aware of these particular symptoms. On the other hand, we found that patients posted less about cognitive complaints than did their relatives or friends, suggesting that patients suffering from cognitive deterioration are often less aware of their condition and less prone to using Twitter. These findings also point to patients' psychopathologies influencing the type of content they publish over social media (52, 53).

Furthermore, the relatively small number of tweets concerning metabolic disturbances posted by patients with schizophrenia is worth highlighting. Psychotic disorders are often associated with altered glucose homeostasis, insulin resistance, hyperlipidemia, and hypertension (54, 55). This is notable because resultant cardiovascular diseases are largely responsible for the shorter life expectancy of people with schizophrenia, which is reduced by more than a decade when compared with that of the general population (54, 55). Our observation of the limited interest in metabolic disturbances shown by patients is consistent with previous studies reporting that people with schizophrenia often possess other risk factors for cardiovascular disease, in addition to the use of antipsychotic medications, including a sedentary lifestyle, a poor diet and smoking (56, 57). Thus, our results emphasize an insufficient awareness of healthy habits by these patients (58). Additionally, it is troubling that neither health professionals nor health institutions have dedicated much attention to the promotion of cardiovascular health, despite metabolic disturbances being responsible for the excessive mortality of these patients (59).

Among the Twitter community, we also investigated the interest generated by tweets related to specific antipsychotic medications. We used the number of retweets and likes generated by each tweet as our standard of measure (37). Our results showed that, with regards to user type, health institutions obtained the highest retweet-to-tweet ratio. Being that they are usually very influential in the medical field, health institutions tend to have a greater number of followers than do personal accounts (60). This trend reflects the importance of having health institutions and professionals involved in health conversations since patient education is the first step in treatment effectiveness, a fact that Twitter users appear to appreciate. In regard to content, those tweets asking for or offering help were the ones most retweeted, further reinforcing the notion of Twitter serving primarily as a community of users (61). Finally, concerning specific medications, the ones that generated the most interest were aripiprazole, levomepromazine, and haloperidol. These results perhaps may be explained by the fact that Twitter is an international platform, as evidenced by the high use of aripiprazole in Europe and the United States, while levomepromazine and haloperidol are commonly employed in other parts of the world for the treatment of schizophrenia. In fact, a majority of tweets, most notably from South America, asked for assistance in acquiring these medications. Probably for their connection to treatment, levomepromazine, and haloperidol were among the two most retweeted and liked medications.

Previous research has suggested that it may be in the best interest of health care providers and the pharmaceutical industry to focus on disease diagnosis and treatment (62, 63). Additionally, it has been noted that some companies have been especially active in promoting the benefits of their products over social media (64, 65). In fact, the food and beverage industries have been increasingly promoting their brands over social media platforms, as well as utilizing posts to advertise them (66). However, according to our results, only a small percentage of the tweets studied related to antipsychotic medications promoted commercial activities. Moreover, paliperidone, despite being one of the latest drugs launched on the market, accounted for only a small number of tweets. As well, the content that obtained the highest retweet-to-tweet ratio was that related to either the asking for or offering of help. These results suggest that Twitter is frequented by users participating in conversations related to antipsychotic medications for the primary purpose of seeking out information and support rather than looking for commercial opportunities.

## Limitations

This study has some limitations. First, since Twitter users tend to be younger than the general population, our findings may not pertain to older age ranges (67, 68). Indeed, younger patients in the earlier stages of psychotic disorders are generally more active, suffer less cognitive impairment and are more likely to engage over social media. Secondly, we were unable to examine how clinical characteristics such as symptom severity, illness duration or cognitive dysfunction found in individuals with psychosis influenced the content of their social media posts due to a lack of psychiatric evaluation. Third, the codebook design and text analysis we used imply a degree of subjectivity. However, this methodology is consistent with previous medical research studies on Twitter and could be applied to assorted topics by various authors (69–71). Fourth, among the list of keywords we included both generic names and brand names but those tweets that included spelling mistakes might have been left out.

## Conclusions

Our results highlight the potential to leverage social media for a better understanding of patients suffering from schizophrenia and their treatment in a manner that is more comprehensive, one that not only includes health care providers and health institutions but also family members, friends, and other social media users. Although guidelines recommend assessing the efficacy of, adherence to and tolerability toward treatment during medical consultations, in the treatment of a patient with schizophrenia, one's individual environment beyond the confines of a doctor's office is extremely important, especially as it can frequently extend to include other influences, many of which are found on social media and are worth analyzing due to the broadened perspective they can offer.

## Data Availability Statement

The raw data supporting the conclusions of this article will be made available by the authors, without undue reservation.

## Ethics Statement

The studies involving human participants were reviewed and approved by the Research Ethics Committee of the University of Alcala (OE 14_2020). Written informed consent for participation was not required for this study in accordance with the national legislation and the institutional requirements.

## Author Contributions

MAA-M, JQ, and MÁ-M: conceptualization, validation, and resources. MAA-M, CD-V, JS-V, LA, RR-J, JQ, and MÁ-M: methodology and data curation. MAA-M, CD-V, JS-V, LA, JG, RS-B, FM, MO, GL, RR-J, JQ, and MÁ-M: formal analysis, investigation writing—original draft preparation, and writing—review and editing. JQ and MAA-M: supervision. MÁ-M: project administration and funding acquisition. All authors have read and agreed to the published version of the manuscript.

## Funding

This work was partially supported by grants from the Fondo de Investigación de la Seguridad Social, the Instituto de Salud Carlos III (PI18/01726) (Spain), the Programa de Actividades de I+D de la Comunidad de Madrid en Biomedicina (B2017/BMD-3804), Madrid (Spain), and Helekulani SL.

## Conflict of Interest

JS-V was employed by IBM. RR-J has been a consultant for, spoken at events of, or received grants from the Instituto de Salud Carlos III, the Fondo de Investigación Sanitaria (FIS), the Centro de Investigación Biomédica en Red de Salud Mental (CIBERSAM), the Madrid Regional Government (S2010/BMD-2422 AGES, S2017/BMD-3740), JanssenCilag, Lundbeck, Otsuka, Pfizer, Ferrer, Juste, Takeda, Exeltis, Casen-Recordati, and Angelini. The remaining authors declare that the research was conducted in the absence of any commercial or financial relationships that could be construed as a potential conflict of interest.

## Publisher's Note

All claims expressed in this article are solely those of the authors and do not necessarily represent those of their affiliated organizations, or those of the publisher, the editors and the reviewers. Any product that may be evaluated in this article, or claim that may be made by its manufacturer, is not guaranteed or endorsed by the publisher.
